# Increased Antimicrobial and Multidrug Resistance Downstream of Wastewater Treatment Plants in an Urban Watershed

**DOI:** 10.3389/fmicb.2021.657353

**Published:** 2021-05-24

**Authors:** Maitreyee Mukherjee, Edward Laird, Terry J. Gentry, John P. Brooks, Raghupathy Karthikeyan

**Affiliations:** ^1^School of Biological, Environmental, and Earth Sciences, The University of Southern Mississippi, Long Beach, MS, United States; ^2^Department of Soil and Crop Sciences, Texas A&M University, College Station TX, United States; ^3^USDA-ARS, Mississippi State, Starkville, MS, United States; ^4^Department of Agricultural Sciences, Clemson University, Clemson, SC, United States

**Keywords:** antimicrobial resistance, antibiotic resistance genes, wastewater treatment plant, antibiotic resistant bacteria, multidrug resistance

## Abstract

Development and spread of antimicrobial resistance (AMR) and multidrug resistance (MDR) through propagation of antibiotic resistance genes (ARG) in various environments is a global emerging public health concern. The role of wastewater treatment plants (WWTPs) as hot spots for the dissemination of AMR and MDR has been widely pointed out by the scientific community. In this study, we collected surface water samples from sites upstream and downstream of two WWTP discharge points in an urban watershed in the Bryan-College Station (BCS), Texas area, over a period of nine months. *E. coli* isolates were tested for resistance to ampicillin, tetracycline, sulfamethoxazole, ciprofloxacin, cephalothin, cefoperazone, gentamycin, and imipenem using the Kirby-Bauer disc diffusion method. Antimicrobial resistant heterotrophic bacteria were cultured on R2A media amended with ampicillin, ciprofloxacin, tetracycline, and sulfamethoxazole for analyzing heterotrophic bacteria capable of growth on antibiotic-containing media. In addition, quantitative real-time polymerase chain reaction (qPCR) method was used to measure eight ARG – *tetA, tetW, aacA, ampC, mecA, ermA, blaTEM*, and *intI1* in the surface water collected at each time point. Significant associations (*p* < 0.05) were observed between the locations of sampling sites relative to WWTP discharge points and the rate of *E. coli* isolate resistance to tetracycline, ampicillin, cefoperazone, ciprofloxacin, and sulfamethoxazole together with an increased rate of isolate MDR. The abundance of antibiotic-resistant heterotrophs was significantly greater (*p* < 0.05) downstream of WWTPs compared to upstream locations for all tested antibiotics. Consistent with the results from the culture-based methods, the concentrations of all ARG were substantially higher in the downstream sites compared to the upstream sites, particularly in the site immediately downstream of the WWTP effluent discharges (except *mecA*). In addition, the Class I integron (*intI1*) genes were detected in high amounts at all sites and all sampling points, and were about ∼20 times higher in the downstream sites (2.5 × 10^7^ copies/100 mL surface water) compared to the upstream sites (1.2 × 10^6^ copies/100 mL surface water). Results suggest that the treated WWTP effluent discharges into surface waters can potentially contribute to the occurrence and prevalence of AMR in urban watersheds. In addition to detecting increased ARG in the downstream sites by qPCR, findings from this study also report an increase in viable AMR (HPC) and MDR (*E. coli*) in these sites. This data will benefit establishment of improved environmental regulations and practices to help manage AMR/MDR and ARG discharges into the environment, and to develop mitigation strategies and effective treatment of wastewater.

## Introduction

Incidences of antimicrobial resistance (AMR) in previously susceptible pathogenic bacteria are on the rise (Jones et al., 2008). Another related issue of serious consequences to public health is the proliferation of multidrug resistance (MDR) within both pathogenic and non-pathogenic bacterial populations (Levy and Marshall, 2004). This has been identified as a critical issue of profound concern by several global organizations such as the World Health Organization, U.S. Center for Disease Control, the National Academy of Science’s Institute of Medicine, the Federal Interagency Task Force on Antimicrobial Resistance, the Infectious Diseases Society of America and numerous other worldwide public health authorities (Spellberg et al., 2008; Allen et al., 2010; Bush et al., 2011; Pruden, 2014). AMR and MDR development in pathogenic bacteria results in several issues concerning public health including limited treatment options, increased morbidity and mortality rates, increased hospital stays, high treatment costs, and the increased necessity for novel antibacterial agents (Goossens et al., 2005; Lim and Webb, 2005; Chopra et al., 2008; Kemper, 2008; Blot et al., 2010; Lye et al., 2012; Nikaido and Pagès, 2012; Naqvi et al., 2013; Worthington and Melander, 2013).

Major contributors to the spread of antibiotic resistance include excessive use in humans and animals, overcrowding and increased rates of transmission between people in communities and hospitals, and the failure of implementing and executing proper hygiene and disinfection practices (Gopal Rao, 2012). AMR and MDR can rapidly spread within bacterial populations of related and unrelated species (Davison, 1999; Pepper and Gentry, 2015) by horizontal gene transfer (Dzidic and Bedeković, 2003) of antibiotic resistance genes (ARG) present in plasmids, transposons, and integrons, or through development of spontaneous mutations (Courvalin, 1994). In recent years, understanding the sources of AMR, MDR and ARG distribution has been deemed critical to eventually control and regulate the spread of ARG (Allen et al., 2010). Yet, enormous gaps still remain in our current knowledge about the occurrence, spread and distribution of AMR, MDR and ARG in the reservoirs found in natural and artificial environments (Allen et al., 2010; Wright, 2010; Rizzo et al., 2013).

While the mechanisms by which antibiotic resistant bacteria (ARB) and ARG are transported and spread through the environment are still not fully understood, previous studies have predicted connections between human activity and the conveyance of resistance traits through agricultural operations, aquatic environments, and sediments (Pei et al., 2006; Baquero et al., 2008; Zhang X. et al., 2009; Huijbers et al., 2015). Pharmaceutical compounds and resistant bacteria may also be introduced to wastewater treatment systems through hospital, industrial, and residential wastewater discharge, and then introduced to the environment (Zuccato et al., 2010; Amador et al., 2015; Verlicchi et al., 2015).

The evolution and development of resistance in clinically important bacteria could be a result of increased opportunities of genetic exchanges with the environmental ARG pool (Bouki et al., 2013; Rizzo et al., 2013; Huijbers et al., 2015, 2019; Chu et al., 2018). Urban WWTPs are increasingly being suspected to be one of the major reservoirs of AMR, MDR and ARG and their mobilization into the environment through effluents (Iwane et al., 2001; Jindal et al., 2006; Szczepanowski et al., 2009; Zhang Y. et al., 2009; Bouki et al., 2013; Korzeniewska et al., 2013; Rizzo et al., 2013; Huijbers et al., 2015; Chu et al., 2018). Contemporary municipal WWTPs are typically incapable of specifically addressing the influx of antibiotics (Adams et al., 2002; Rizzo et al., 2013). Wastewater treatment has also been found to be generally ineffective against certain strains of resistant enterococci, specifically with resistance to ciprofloxacin, erythromycin, and tetracycline (da Silva et al., 2006), with the prevalence of ciprofloxacin resistance actually increasing through the treatment process. The presence of sulfonamide resistance genes in a river environment was found to increase significantly downstream of a swine feedlot WWTP (Hsu et al., 2014). Iwane et al. (2001) found that *Escherichia coli* isolates obtained along the Tama River in Tokyo, Japan expressed increasing resistance to antibiotic agents as sampling moved downstream, and was attributed to treatment plant discharge. Studies tend to vary with respect to the efficiency in which resistant organisms are removed during the treatment process, the microbial species expressing resistance in the effluent, and the antimicrobial agents to which the organisms express resistance. It also should be noted that different WWTP unit operations will affect the overall removal efficiency and ultimately antibiotic resistant bacteria discharge to the environment (Sayah et al., 2005; Janezic et al., 2013; Hamilton et al., 2020). Czekalski et al. (2012) found that while WWTPs reduced total bacterial loads in the effluent, there was an observed increase in multidrug resistant bacteria and ARG which were then found to accumulate in the sediment of the plant outlet. *Aeromonas* and *Pseudomonas aeruginosa* isolates obtained from some water reservoirs were found to express 50 and 100% multi-drug resistance, respectively (Blasco et al., 2008). A recent review also suggests the importance of accounting for stormwater as a key source of ARG propagation considering the cumulative impact stormwater runoff may have as it comes in contact with overflows from untreated wastewater, sewer and sanitary discharges, among others (Hamilton et al., 2020). Understanding of the impacts of urbanization and wastewater effluent on the presence of antibiotic resistance in the environment will aid in future efforts to address antibiotic resistance through treatment plant process design - establishing more informed guidelines and proper regulations surrounding WWTP practices. We investigated the relationship between urban development and the occurrence and persistence of antimicrobial resistance in the surrounding aquatic environment using antimicrobial resistance data produced by culture-based and quantitative PCR methods. In this study, we collected surface water samples from six sites in an urban watershed – upstream and downstream of two wastewater treatment plants in the Bryan - College Station (BCS), Texas, over a period of nine months. Heterotrophic bacteria capable of growing on antibiotic amended media (HPC-Ab) and *E. coli* were isolated from the six sampling sites and evaluated for resistance to selected antibiotics. Surface water samples were also analyzed and quantified for the presence of eight different ARG targets. Rates of antimicrobial resistance for *E. coli* isolates and antimicrobial resistant heterotrophic communities were compared by sampling site and their relative position with respect to WWTPs to determine if WWTP discharge may affect the antimicrobial resistance profiles of surface water bacteria in the surrounding environment.

## Materials and Methods

### Study Area

**FIGURE 1:**
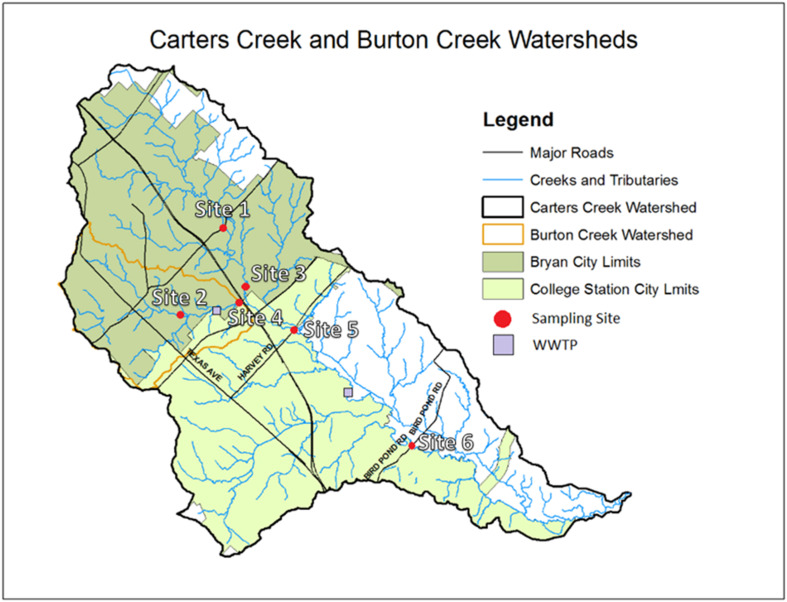
Map of the Carters Creek Watershed and locations of the six sampling sites and the two WWTPs.

Six sampling sites were established within the boundaries of the Carters Creek watershed in BCS area on the main stems of Carters Creek and Burton Creek (Figure 1). Sites were selected to represent areas up and downstream of two WWTPs. Sites 1, 3, 5, and 6 were located on the main stem of Carters Creek, and sites 2 and 4 located on the main stem of Burton Creek. Most sites (all but site 3) were located at the intersection of the respective creek and an overpassing bridge. Sites 2, 4, 5, and 6 were sampled upstream of the bridge crossing, and site 1 was sampled directly underneath the overpass. Site 3 was sampled on the stream stem of Carters Creek running adjacent to the highway, upstream of its confluence with Burton Creek. Site 2 was located at the outlet of a channelized stretch of Burton Creek, characterized by shallow flow with substantial algal growth on the concrete surface. All of the sampling sites selected in this study are regular water quality monitoring sites for the Texas Commission on Environmental Quality (TCEQ, 2006) since the commencement of an ongoing Carters Creek watershed Total Maximum Daily Load (TMDL) project in August, 2007.

The Burton Creek WWTP is located upstream and Carters Creek WWTP downstream. Burton Creek WWTP permitted discharge is 8 MGD. Carters Creek WWTP was built in the late 50’s to treat about 0.6 MGD to meet the population of 6,000 people. Now the treatment capacity is 9.5 MGD, even though it reaches only about 7 MGD maximum. Both the treatment plants use activated sludge process to remove carbonaceous and nitrogenous BOD. They both do not use any tertiary treatments. Burtons Creek WWTP uses chlorination to remove pathogens while Carters Creek WWTP applies UV as a disinfection method. Dewatered sludge is digested anaerobically at Burton Creek and aerobic digestion is used in Carters Creek. To meet the population growth another 2 MGD plant was constructed (Lick Creek WWTP) and in operation since early 90’s. Additional information on this watershed and the water quality standards and compliance is available at https://www.tceq.texas.gov/waterquality/tmdl/85-carterscreek.html. Urbanization-related maps are provided in Supplementary Figure 4. Specific information concerning the sampling sites is included in Supplementary Table 1 (see Supplementary Material).

### Sample Collection

A total of six separate sampling events were conducted over a nine-month period between July 2015 and April 2016. Surface water samples were collected using ∼500 mL Whirl-Pak^®^ sterile bags (eNasco, Fort Atkinson, WI, United States) attached to a sampling pole. Water samples were collected from the mid-point of the stream flow approximately 3 cm below the surface. Samples were immediately transferred on ice back to the laboratory and processed within 6 h of collection.

### *Escherichia coli* Isolation and Antibiotic Susceptibility Testing

Inhibition zone diameters were measured and recorded in millimeters and compared to CLSI (Clinical and Laboratory Standards Institute) standards to determine if each isolate was susceptible or resistant to each antibiotic. Isolates were subsequently confirmed as *E. coli* through PCR amplification of *uidA* with *E. coli*-specific primers (data included in Supplementary Material).

Initially, four concentrations of each water sample were prepared (1.0, 0.1, 0.01, and 0.001) by ten-fold serial dilutions in phosphate-buffered saline solution (PBS). Ten mL of each dilution was then filtered through a 0.45 μm filter membrane (Millipore, Billerica, MA, United States) by vacuum filtration. Filter membranes were placed on 47 mm Difco^®^ Modified mTEC agar plates (Becton, Dickinson and Company, Sparks, MD, United States) and incubated at 35°C for 2 h and then 44.5°C for 24 h in accordance with EPA Method 1603 (USEPA, 2005). Following incubation, ten presumed *E. coli* (magenta) colonies for each of the six sites were randomly selected, transferred to Difco^®^ Tryptic Soy agar (Becton, Dickinson and Company, Sparks, MD, United States) using a sterile loop, and incubated at 35°C for 24 h. *E. coli* cell suspensions were prepared by transferring two colonies of each isolate into tubes with 5 mL of BBL^®^ Tryptic Soy Broth (Becton, Dickinson and Company, Sparks, MD, United States) and incubating at 35°C for 3 h while shaking at 150 rpm. Tubes were checked for turbidity against a pre-prepared 0.5 McFarland standard corresponding to a 10^7^–10^8^ CFU/mL bacterial cell count in the broth.

After incubation, sterile swabs were used to inoculate 100 mm Mueller Hinton Agar (MHA) plates (Neogen Corporation, Lansing, MI, United States). Antibiotic resistance of the *E. coli* isolates was determined by the Kirby-Bauer method for antibiotic susceptibility (Bauer et al., 1966). Eight antibiotic susceptibility discs (Becton, Dickinson and Company, Franklin Lakes, NJ, United States) of tetracycline (30 μg), ampicillin (10 μg), ciprofloxacin (5 μg), imipenem (10 μg), sulfamethoxazole/trimethoprim (23.75/1.25 μg), gentamicin (120 μg), cefoperazone (75 μg), and cephalothin (30 μg) were stamped onto each MHA plate using a BBL^®^ Sensi-Disc^®^ 8-place Dispenser (Becton, Dickinson and Company, Franklin Lakes, NJ, United States). The MHA plates were then incubated at 35°C for 16–24 h and the diameters of the inhibition zones measured to determine resistance or susceptibility of each isolate to the antibiotics according the Clinical and Laboratory Standards Institute (CLSI) standards. Control organisms, *E. coli* 25922, *Staphylococcus aureus* 25923, and *Pseudomonas aeruginosa* 27852, were used to ensure consistency during the antibiotic disc diffusion process.

### PCR Isolate Confirmation

PCR amplification of the *E. coli* specific *uidA* sequence was used to confirm all isolates collected as *E. coli* (Bower et al., 2005). Cell suspensions of each presumed *E. coli* isolate were prepared by suspending bacterial growth from the MHA agar in 100 μL of sterile, distilled water. PCR mixtures (50 μL) were prepared consisting of 25 μL of GoTaq^®^ G2 Green Master Mix (Promega, Madison, WI, United States), 1.75 μL (350 nM) each of the forward (uidA1318F) and reverse (uidA1698R) primers (Integrated DNA Technologies, Coralville, IA, United States), 5 μL of cell suspension as the template DNA, and 16.5 μL of sterile nuclease-free water. *E. coli* 25922 isolates were used for the positive control. Primer sequences, target, and reference are shown in Table 1A.

**TABLE 1:** A. PCR and B. qPCR primers and conditions used in this study.

Target	Primer sequence (Forward-F, Reverse-R)	Amplicon size and Annealing temperature	References
***A.* A. PCR conditions**			
*E. coli* β –glucuronidase	F- 5′CCGATCACCTGTGT CAATGT 3′ 5′GTTACCGCCAACGCGC AATA 3′	400 bp 60°C	Bower et al., 2005
***B.* B. qPCR conditions**			
Class I integron (*intI1*)	F- CTGGATTTCGATCACGG CACG R- ACATGCGTGTAAATCAT CGTCG	473 bp 60°C	Hardwick et al., 2008
Tetracycline (*tetA*)	F- GCTACATCCTGCTTGC CTTC R- CATAGATCGCCGTGA AGAGG	210 bp 62°C	Fan et al., 2007
Tetracycline (*tetW*)	F- GAGAGCCTGCTATATG CCAGC R- GGGCGTATCCACAAT GTTAAC	168 bp 64°C	Aminov et al., 2001
Ampicillin (*ampC*)	F- TTCTATCAAMACTG GCARCC R- CCYTTTTATGTACCC AYGA	550 bp 55°C	Schwartz et al., 2003
Erythromycin (*ermA*)	F- GAAATYGGRTCAGGAA AAGG R- AAYAGYAAACCYAAA GCTC	332 bp 55°C	Chen et al., 2010
Methicillin (*mecA*)	F- AAAACTAGGTGTTGGTGA AGATATACC R- GAAAGGATCTGTACTGG GTTAATCAG	146 bp 55°C	Sabet et al., 2007
Aminoglycoside (*aacA*)	F- TCCTTACTTAATGACCG ATGTACTCT R- TCTTCGCTTTCGC CACTTTGA	146 bp 55°C	Sabet et al., 2007
β-lactamase (*blaTEM*-Univ)	F- CACTATTCTCAGAATGA CTTGGT R- TGCATAATTCTCTTACTG TCATG	84 bp 60°C	Lachmayr et al., 2009

PCR conditions included one initial heating cycle at 94°C for 4 min; followed by 35 cycles at 94°C for 30 s, 60°C for 30s, and 72°C for 30 s; a final cycle at 72°C for 6 min, and then held at 4°C. DNA electrophoresis was performed in a 2% agarose gel (Amresco, Solon, OH, United States) stained with ethidium bromide (Sigma-Aldrich, St. Louis, MO, United States) and a 1X Tris-Borate-EDTA (TBE) (Fisher BioReagents, Fair Lawn, NJ, United States) buffer solution. A 100 bp ExACTGene^TM^ DNA ladder (Fisher BioReagents, Fair Lawn, NJ, United States) was used as the marker (Data in Supplementary Material, Section “PCR Isolate Confirmation Results” and Supplementary Figure 1).

### Heterotrophic Plate Counts

Heterotrophic bacteria capable of growth on antibiotic-containing media was analyzed using the following protocol. This method potentially captures both intrinsic and acquired resistant bacteria, but does not differentiate between the populations. Four ten-fold serial dilutions of each of the six water samples were prepared (1.0, 0.1, 0.01, and 0.001) by diluting in PBS. Thirty microliters of each dilution were spread-plated onto five sets of 47 mm plate Bacto^®^ Reasoner’s 2A (R2A) agar (Difco Laboratories, Detroit, MI, United States) amended with the following antibiotics: 32 μg/mL ampicillin (HPC-Am) (Ward’s Science, Rochester, NY, United States), 16 μg/mL tetracycline (HPC-Te) (Alfa Aesar, Ward Hill, MA, United States), 4 μg/mL ciprofloxacin (HPC-Cpr) (TCI America, Portland, OR, United States), 50.4 μg/mL sulfamethoxazole (HPC-Su) (Chem-Impex International Inc., Wood Dale, IL, United States), and un-amended R2A (HPC) with no antibiotic. Antibiotic concentrations in the agar were determined based on prior studies and are generally around half the strength of either the IV or oral dosage concentrations (Pei et al., 2006; Gao et al., 2012; Garcia-Armisen et al., 2013). Plates also contained 200 μg/mL of cycloheximide (Amresco, Solon, OH, United States) as a fungicide to suppress any fungal growth. All plates were incubated at 28°C for 5 days before obtaining bacterial CFU plate counts. The limit of detection was one CFU in 30 μL of undiluted sample, or 1.52 log_10_ CFU/mL. There were five instances in which no bacteria were culturable within the sample volume and concentration limit; four of the ciprofloxacin-amended plates, and one of the tetracycline-amended plates. These results were reported as below the limit of detection, and were represented as ½ the limit of detection (16.67 CFU/mL) for statistical analysis.

### DNA Extraction and Quantification

From each sample, 100 mL of water was filtered through sterile 0.22 μm Millipore membrane filters of 47 mm diameter and stored in sealed sterile petri plates with the biomass facing up at −80°C for further analyses. The environmental DNA was extracted from these membrane filters using a MoBio PowerWater^®^ DNA Isolation Kit (currently Qiagen, Germantown, MD, United States), following the manufacturer’s instructions. Biomass from the filters were prepared and lysed using the PowerWater^®^ bead tubes on a MoBio vortex adapter. For DNA extraction, the centrifugation method was used to bind DNA onto the provided spin filter, washed and finally eluted using the kit’s elution buffer. The final elution volume for each DNA sample was 100 μl. The DNA thus obtained was analyzed for concentration using a NanoDrop spectrophotometer (Thermo Fisher Scientific) and used for further quantitative PCR analysis.

### Quantitative PCR Analysis

The DNA from each sample was analyzed for the presence of eight ARG: *intI1, tetA, tetW, ampC, blaTEM, mecA, aacA*, and *ermA*. The primer pairs and conditions used for each qPCR analysis is listed in Table 1. Each primer pair was tested with each qPCR standard for accuracy of product size and annealing temperature by confirming with end point PCR and agarose gel electrophoresis before using for final qPCR analyses. For qPCR analysis, each sample was run in duplicate. Each standard (standard detail listed in Supplementary Table 2, see Supplementary Material) was prepared by (i) extracting DNA from the control cultures using the MoBio microbial DNA isolation kit (currently Qiagen, Germantown, MD), (ii) PCR amplification using respective primers, (iii) confirming purity and amplicon size using agarose gel, and (iv) purifying using the Qiagen PCR purification kit (Germantown, MD, United States). The standard DNA was then quantified using a NanoDrop spectrophotometer (Thermo Fisher Scientific) and serially diluted to generate qPCR standard curves for each primer pair. Each qPCR reaction had a final volume of 25 μL and consisted of the following reagents: 12.5 μL of 2X SYBR Green (Applied Biosystems), 1.25 μL of 10 μM of each primer, and 10 μL of environmental DNA from each site. The reactions were run in duplicates on 96-well Eppendorf green-skirted plates (Fisher Scientific), and sealed with a plate sealer using qPCR sealing films (BioRad) before analysis using an Eppendorf Realplex2 Mastercycler system. The results were analyzed using the Eppendorf Realplex^2^ software and converted into genomic units per 100 mL (GU 100 mL^–1^) of water sample (Brooks et al., 2014).

### Statistical Analyses

*Escherichia coli* isolate responses to antibiotic susceptibility disc diffusion were categorized as either susceptible or resistant (including intermediate resistance) and assigned a binary value for each response: 1 for resistant and 0 for susceptible. Then, isolates and isolate responses were grouped into a number of various categories and tested for significant associations by chi-square analysis. Groupings were generally done by pairing binary data from two individual sampling sites or two groups of sampling sites, generating two-by-two grids with one degree of freedom. Significant differences were determined by Chi square sums of 3.84 or greater, or *p* < 0.05 for one degree of freedom. Post hoc multi-comparison tests were carried out for sample site, where appropriate, by conducting pairwise Chi square tests with Bonferonni adjusted p-values. Statistical analysis of the HPC-Ab and box plot generation was done using SAS^®^ University Edition (Cary, NC, United States). Significant differences in the abundance and normalized resistance rates of heterotrophic ARB were evaluated using one-way ANOVA by least-significant-difference (LSD) comparison. Significant differences were checked for homogeneity of variance by Levene’s test. In cases where significant differences in homogeneity were found in the data set, it was (then) determined by Welch’s ANOVA. Relationships were considered to be significant at *p* < 0.05.

Antibiotic resistance gene levels per 100 mL (GU 100^–1^ mL) were log_10_ transformed prior to statistical analysis. Differences in response variables (stream position and creek) was assessed for all measured ARG using the mixed procedure in SAS Enterprise Guide 7.1 (SAS Institute). Creek was used as a random variable in the mixed model. Residuals were normally distributed and means were post-hoc adjusted and compared using least square means. All differences were significant at *p* < 0.05, unless otherwise noted.

## Results

### *E. coli* Resistance Patterns to Individual Antibiotics

**FIGURE 2:**
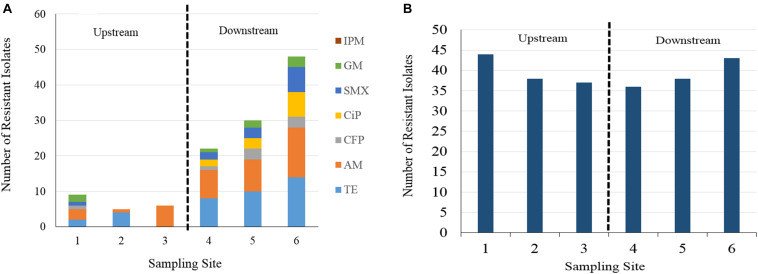
**(A)** Number of resistant isolate responses to seven of eight antibiotics at different sampling site by Kirby Bauer disc diffusion method. Cephalothin is excluded for visibility of less frequently occurring AB resistances. IPM, imipenem, GM, gentamycin, SMX, sulfamethoxazole, CiP, ciprofloxacin, CFP, cefoperazone, AM, ampicillin, TE, tetracycline. **(B)** Column chart of cephalothin resistant *E. coli* isolate responses. Resistance occurred at a greater frequency than other antibiotic resistances, more consistently across upstream and downstream sampling sites.

The number of isolates expressing resistance to individual antimicrobial agents by sampling site are displayed in Figure 2 and Supplementary Table 3. Twelve percent of all isolates were susceptible to all 8 antibiotics. A large proportion (84%) of all isolates expressed resistance to cephalothin, with rates of resistance at each individual sampling site falling consistently between 77 and 90% of the isolates collected. The next highest rates of resistance after cephalothin occurred with ampicillin and tetracycline at 15 and 14% of all isolates, respectively. Resistance to ampicillin was expressed in 41 isolates, resistance to tetracycline was expressed in 38 isolates, and resistance to cefoperazone, gentamycin, ciprofloxacin, and sulfamethoxazole/trimethoprim was found in a fewer number of isolates, at rates of 3, 3, 4, and 5%, respectively. All 280 isolates were susceptible to imipenem.

A column chart of isolate resistance responses by sampling site and antibiotic shows an increase in the total number of resistant responses in the downstream sampling sites (Figure 2). Isolates collected from the downstream sampling sites expressed resistance more frequently and to diverse antimicrobial agents than the upstream sites. Sampling site 1 displays the most diversity in resistance to different agents in the upstream group, due to one isolate sampled during event two expressing resistance to six agents. The number of total resistant responses also appears to increase as the sampling sites are farther away downstream. For ampicillin, resistance rates fell between 2 and 13% for isolates obtained upstream of WWTP discharges and 17–28% for isolates obtained downstream of WWTP discharges. For tetracycline, resistance rates fell between 0 and 9% for isolates obtained upstream of WWTP discharges and 17–28% for isolates obtained downstream of WWTP discharges. Resistance to cefoperazone, gentamycin, ciprofloxacin and sulfamethoxazole were found more frequently in the isolates obtained from downstream sampling sites. Gentamycin resistance was the only instance in which isolate resistance was found to occur more frequently in one of the upstream sites than in one of the downstream sites (site 1 vs. site 4). Cephalothin resistance is presented separately in Figure 2B as to not visually overwhelm the less frequently occurring antibiotic resistances. Cephalothin resistance occurred at a greater frequency and more consistently across all sampling sites than the observed resistance to other antibiotics, irrespective of proximity to WWTP.

Chi-square tests for isolate resistance by individual sampling site (Supplementary Figure 2) showed significant differences (*p* < 0.003) between at least one pair of sites for ampicillin, sulfamethoxazole, tetracycline, and ciprofloxacin. The majority of these occurred between site pairings in which one site was upstream of a WWTP and the other site was downstream of a WWTP. Only one test reported a significant difference between two sites with the same relative location to a WWTP. This result was reported for the rate of isolate resistance to tetracycline between sites 2 and 3, corresponding to the Burton Creek site upstream of the WWTP and the Carters Creek site upstream of its confluence with Burton Creek, respectively. When sampling sites were categorized into either an upstream (sites 1–3) or downstream (sites 4–6) group, a significant difference (*p* < 0.05) was found to exist in isolate rates of resistance to ampicillin, tetracycline, cefoperazone, ciprofloxacin, and sulfamethoxazole. While cefoperazone resistance did not increase significantly between any individual sampling sites, there was a significant increase when rates of isolate resistance were categorized and compared between the upstream and downstream.

### *E. coli* Multi-Drug Resistance Patterns

Binomial resistance values determined by the number of resistant responses of each isolate were tallied, organized by sampling site, and sorted into five groups – isolates resistant to 1, 2, 3, or ≥4 agents (Table 2). Out of the 280 isolates, the majority (88%) showed resistance to at least 1 antibiotic agent. A total of 28 isolates (10% of total) showed resistance to 2 agents, 9 (3% of total) showed resistance to 3 agents, and 17 (6% of total) showed resistance to 4 or more agents. Resistance responses were also sorted by type of antibiotic and number of agents that each isolate was resistant to (Table 3). Cephalothin resistance was again the most frequently occurring (95%) antibiotic resistance in the sample set of multi-drug resistant isolates (resistant to two or more agents). Out of all isolates that were resistant to at least one antibiotic, 74% were only resistant to cephalothin, and cephalothin resistance accounted for 95% of all single-drug resistant isolates. Isolates only resistant to tetracycline, ampicillin, or cefoperazone were found sparingly, each representing less than 2% of the single-drug resistant isolates. No isolates were only resistant to ciprofloxacin, sulfamethoxazole, gentamycin, or imipenem. Isolates resistant to two or more agents were generally resistant to cephalothin and either tetracycline (41%), or ampicillin (48%). Resistance to three agents occurred less frequently than resistance to four or more agents, at only 4% of resistant isolates. All isolates showing resistance to 4 or more antibiotics were resistant to cephalothin, over 80% of these isolates were also resistant to tetracycline, and 90% to ampicillin. Sulfamethoxazole resistance was only found in isolates resistant to three or more agents. Resistance to cefoperazone, ciprofloxacin, sulfamethoxazole, and gentamycin was generally accompanied by several other resistances (Table 3).

**TABLE 2:** Percentage (%) of multi-drug resistant *E. coli* isolates by sampling site.

Site number	Number (% by site) of Isolates with Resistance to *n* agents:					Total
	
	*n* = 0	*n* = 1	*n* = 2	*n* = 3	*n* ≥ 4	
1	5 (10)	39 (80)	4 (8)	0 (0)	1 (2)	49
2	5 (11)	35 (80)	4 (9)	0 (0)	0 (0)	44
3	7 (15)	35 (76)	4 (9)	0 (0)	0 (0)	46
4	8 (17)	29 (62)	5 (11)	2 (4)	3 (6)	47
5	6 (14)	24 (55)	6 (14)	3 (7)	5 (11)	44
6	2 (4)	31 (62)	5 (10)	4 (8)	8 (16)	50
All sites	33 (12)	193 (69)	28 (10)	9 (3)	17 (6)	280

**TABLE 3:** Number of *E. coli* isolates expressing resistance to each antibiotic, grouped by the number of agents the isolate was resistant to.

Antibiotic	Number (%) of Resistant Isolates when Isolate is Resistant to:				Total
	
	1 agent	2 agents	3 agents	≥4 agents	
				
	*n* = 193	*n* = 29	*n* = 9	*n* = 17	*n* = 248
Tetracycline	4 (2)	12 (41)	8 (89)	14 (82)	38 (15)
Ampicillin	3 (1.5)	14 (48)	8 (89)	16 (94)	41 (17)
Cefoperazone	1 (0.5)	0 (0)	0 (0)	7 (41)	8 (3)
Ciprofloxacin	0 (0)	2 (7)	0 (0)	10 (59)	12 (5)
Sulfamethoxazole/Trimethoprim	0 (0)	0 (0)	3 (33)	13 (76)	13 (5)
Gentamycin	0 (0)	1 (3)	1 (11)	6 (35)	8 (3)
Cephalothin	184 (95)	28 (97)	7 (78)	17 (100)	236 (95)
Imipenem	0 (0)	0 (0)	0 (0)	0 (0)	0 (0)

Of the 54 multi-drug resistant isolates collected (resistant to at least 2 agents), 41 (76%) were obtained from downstream sites (sites 4–6). All isolates resistant to 3 agents and all but one of the isolates that were resistant to four agents were collected from one of the downstream sites.

Chi-square analysis revealed significant associations between several sampling site pairings for when isolates were classified according to multi-drug resistance (Supplementary Figure 3). The most significant factor contributing to differences in isolate multi-drug resistance was associated when isolates were grouped according to relative upstream and downstream position. A significant association (*p* < 0.001) was found to exist between the number of isolates expressing resistance to 1, 2, 3, and ≥4 antibiotic agents and whether the isolate was collected upstream of any WWTP (sites 1, 2, and 3) vs. downstream of at least 1 WWTP (sites 4, 5, and 6).

### Heterotrophic Plate Counts and HPC-Ab Populations

**FIGURE 3:**
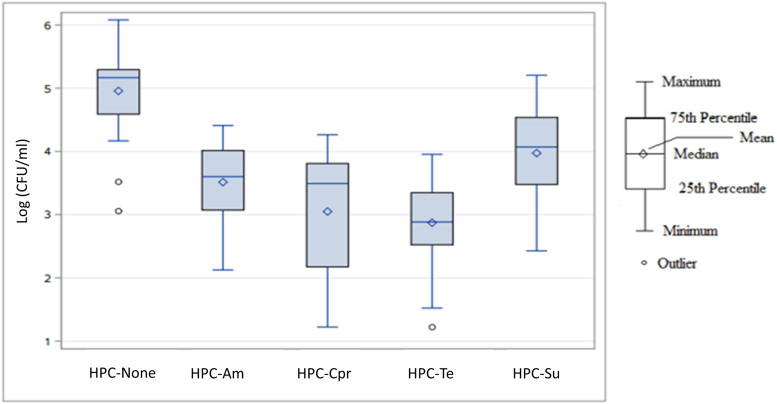
Box plot of log-transformed concentration distributions (log_10_ CFU/mL) of heterotrophic bacteria capable of growing on antibiotic amended agar by antibiotic agent across all sampling events and sampling sites. HPC-none, no antibiotic added; HPC-Am, HPC on ampicillin amended R2A plate; HPC-Cpr, HPC on ciprofloxacin amended R2A plate; HPC-Te, HPC on tetracycline amended R2A plate; HPC-Su, HPC on sulfamethoxazole amended R2A plate.

Heterotrophic bacterial plate counts were obtained during six sampling events to examine the antibiotic resistance profiles of the culturable, HPC-Ab community in the watershed. The log-transformed bacterial concentrations of each treatment category for all sampling events and sampling sites are displayed in Supplementary Table 4 and Figure 3.

For the total concentrations of each subset of heterotrophic bacterial populations, the R2A agar with no antibiotic produced the highest overall concentration with a median value of 1.47 × 10^5^ CFU/mL and a mean value of 1.68 × 10^5^ CFU/mL (Figure 3). Sulfamethoxazole HPC-Su were the next highest with a median concentration of 1.18 × 10^4^ CFU/mL, followed by HPC-Am and HPC-Cpr with median concentrations of 4.00 × 10^3^ CFU/mL and 3.10 × 10^3^ CFU/mL, respectively. HPC-Te had the lowest overall concentration in the study area with a median concentration of 7.67 × 10^2^ CFU/mL. Variance in the total population for each treatment was considerably large, with standard deviations larger than the mean values.

**FIGURE 4:**
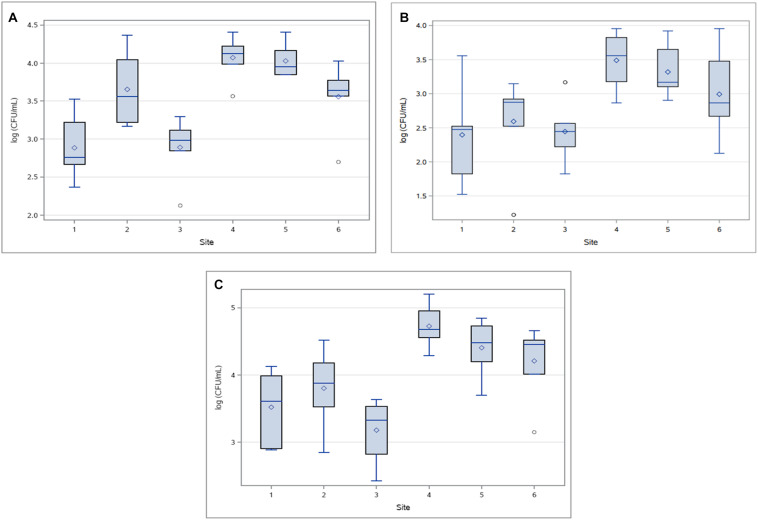
Concentrations (log_10_ CFU/mL) of **(A)** HPC-Am, **(B)** HPC-Tet and **(C)** HPC-Su across sampling sites for all sampling events.

Abundance of HPC-Am varied significantly between sampling sites (*p* < 0.0001), primarily due to the variance occurring between sites 1, 2, 3, and 6 compared to sites 4 and 5 (Figure 4A). HPC did not vary significantly by sampling event (*p* > 0.65). HPC-Am were found in significantly greater (*p* < 0.0001) concentrations in the downstream group relative to WWTP discharge, with mean concentrations of 1.3 × 10^4^ and 1.2 × 10^4^ CFU/mL from sites 4 and 5, respectively.

HPC-Te produced a few outliers due to an atypically compact distribution of concentrations at site 2 (Figure 4B), in contrast to an otherwise expansive distribution and large standard deviations as seen in the other treatments. Standard deviations of HPC-Te concentrations at sites 2 and 3 were lower by one order of magnitude or more than what was typically seen in other resistant bacterial population. The abundance of HPC-Te varied significantly by sampling site (*p* < 0.007) and sampling event (*p* < 0.02), mainly due to considerably higher concentrations sampled during event 6.

HPC-Su were the most prominent across all sampling sites in this study with the highest mean concentration of resistant bacteria at any sampling site of 6.67 × 10^4^ CFU/mL (Figure 4C). Sampling site had a significant influence (*p* < 0.0001) on the concentration of HPC-Ab, mainly due to consistently higher concentrations found at sites downstream from WWTPs.

The mean concentrations HPC-Ab obtained upstream of a WWTP in the tetracycline and sulfamethoxazole amended media were an order of magnitude below the mean concentrations in their respective downstream sites. Significant differences in the abundance HPC-Ab were found to exist between upstream and downstream sites for both the tetracycline (*p* < 0.0001) and sulfamethoxazole amended media (*p* < 0.0001).

### Quantitative Monitoring of Antibiotic Resistance Gene Prevalence and Distribution

**FIGURE 5:**
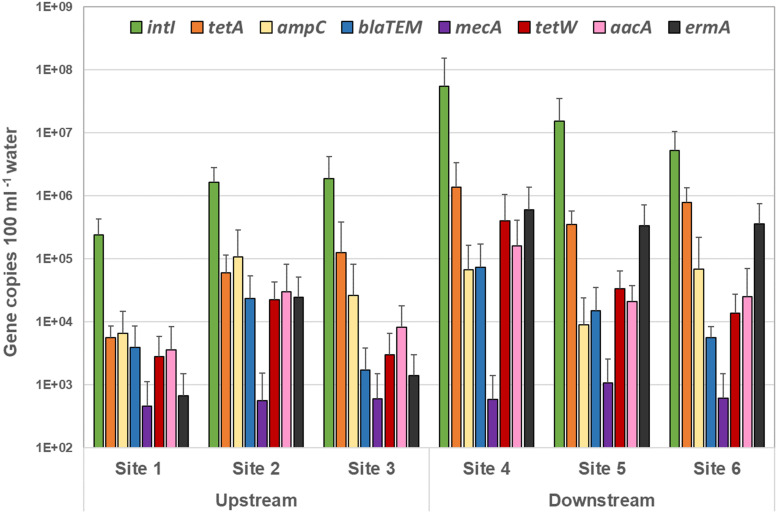
Quantity of each antibiotic resistance gene over all sampling events detected by quantitative PCR in the upstream sites and downstream sites. The target gene values (gene copies 100 ml^–1^ sample) are displayed in mean of all sampling events, and the standard deviations represent the variation among the six sampling events.

Overall, analysis of the frequency, distribution, and quantity of the tested ARG reveal a significantly higher measure of ARG in the sites downstream of the WWTP discharge than that found in the upstream sites (Figure 5). Overall, *blaTEM*, *ermA*, *intI1*, *tetA*, and *tetW* were significantly greater in downstream compared with upstream sites (*p* < 0.05). Except for *mecA, aacA, emrA*, and *blaTEM* genes at specific sites and sampling events, all other ARG were detected in all sites at all location at all sampling events (Figure 5).

Overall, the highest level of ARG was associated with the combined tetracycline resistant genes (*tetA* and *tetW*) – averaged at 7.3 × 10^4^ and 9.9 × 10^5^ copies100 mL^–1^ surface water in the upstream and downstream sites, respectively (Figure 5 and Supplementary Table 5).

A significantly high concentration of the integrase (*intI1*) gene was noted amongst all sites – 1.25 × 10^6^ and 2.5 × 10^7^ gene copies 100 mL^–1^ surface water in the upstream and downstream sites respectively (*p* < 0.0001) (Supplementary Table 5 and Figure 5). In addition, copies of all ARG tested in this study (except for *mecA*) were found to be considerably greater in site 4 – the site immediately downstream of the BCWWTP (Burton Creek Wastewater Treatment Plant) (Supplementary Table 5). This is a notable result and may suggest a role of the WWTP in dissemination of the ARG into the immediate surrounding environment. Additionally, the high prevalence in ARG copies within the immediate downstream site taken together with 40 times higher prevalence of *intI1* genes in the same site (Supplementary Table 5) also suggest that the WWTP may provide a favorable setting for genetic exchanges that lead to development of AMR and MDR in resident bacterial populations.

Among the other tested ARG, erythromycin resistant genes (*ermA*) were found to be substantially high in all downstream sites (4.3 × 10^5^ copies 100 mL^–1^ water) (*p* < 0.0001), particularly high in site 4 (6 × 10^5^ copies 100 mL^–1^ water) – 48 times higher when compared to the upstream sites (9 × 10^3^ copies 100 mL^–1^ water) (Supplementary Table 5). A similar trend, though not significant, was noticed with the broad-spectrum aminoglycoside resistance gene target (*aacA*) – 1.4 × 10^4^ copies 100 mL^–1^ water compared to 1.6 × 10^5^ in site 4 (Supplementary Table 5). Similarly, copies of *blaTEM* genes targeting the TEM type β-lactamases (Bradford, 2001) were significantly greater downstream compared with upstream sites (*p* = 0.0115), for instance, in site 4 (7.33 × 10^4^ copies 100 mL^–1^ water) compared to the combined average of all upstream sites (9.6 × 10^3^ copies 100 mL^–1^ water) (Supplementary Table 5).

## Discussion

### Antimicrobial Resistance to Specific Antibiotics

Ampicillin, sulfamethoxazole, and ciprofloxacin, or closely related drugs (amoxicillin), are among the top 5 antibiotics prescribed for use for adults in the United States (Shapiro et al., 2013; Van Boeckel et al., 2014; Hicks et al., 2015), and all have been found to occur in WWTPs in varying concentrations and design conditions (Batt et al., 2007). In the current study, a significant association (*p* < 0.05) was found to exist between the location of sampling sites relative to WWTPs (upstream group vs. downstream group) and isolates expressing resistance to ampicillin, ciprofloxacin, cefoperazone, sulfamethoxazole, and tetracycline. This supports the hypothesis that WWTP effluent may be contributing to the conveyance of antibiotic resistant bacteria downstream from discharge points. The absence of significant associations between rates of isolate resistance among upstream sites indicates that these differences are not solely dependent on variations between all sampling sites, but also their relative location to WWTP discharge points.

The occurrence of antibiotic resistance may not always imply an outside effect and can be an intrinsic property of the natural environment. In this study, cephalothin represented the highest rate (84%) of resistance found in all isolates, irrespective of their collection location. The high rate of resistance to cephalothin and the ubiquity of its presence suggests that this resistance trait may be partially due to natural occurrence in the watershed. This is consistent with results from other studies performed with *E. coli* isolates obtained from surface waters in Michigan and Illinois, rates of isolate resistance to cephalothin at 80.6 and 80%, respectively (Sayah et al., 2005; Janezic et al., 2013). Cephalothin resistance also represented 95% of the 193 isolates resistant to only 1 antibiotic, dramatically inflating the abundance of isolates classified as resistant to at least 1 antibiotic. If cephalothin was excluded from the AMR data for this study, an additional 184 isolates (66% of total) would be classified as susceptible to all agents.

The rate of isolate resistance to tetracycline (14% of all isolates) was found to be lower than expected when compared to similar research (Jindal et al., 2006; Rajić et al., 2006; Brooks and McLaughlin, 2009; Sullivan and Karthikeyan, 2012). Previous studies have found the occurrence of tetracycline resistance to be prevalent in watersheds associated with agricultural and animal feed lot operations (Jindal et al., 2006; Rajić et al., 2006), with resistance rates of over 90% found in *E. coli* isolates obtained from swine lagoon effluent (Brooks and McLaughlin, 2009). Sullivan and Karthikeyan (2012) reported the prevalence of tetracycline resistant bacteria and tetracycline resistant genes in sediment and surface water samples collected from Carters Creek watershed; however, the majority were found in greater abundance bound in stream sediment samples than in surface water samples. In another study on the Carters Creek watershed, Sullivan and Karthikeyan (2012) found that while the occurrence of tetracycline resistance genes increased downstream of WWTPs, concentrations of tetracycline resistant bacteria were not significantly affected.

Imipenem is a group 2 carbapenem generally reserved as the last line of defense against particularly resilient Gram-negative pathogens and not widely prescribed (Nicolau et al., 2012). Out of all the isolates collected from all sampling sites, all 280 *E. coli* isolates were susceptible to imipenem. Imipenem resistance was not detected in the surface water bacteria of the Carters Creek watershed.

### Multidrug Resistance

A substantial fraction (19%) of all 280 *E. coli* isolates expressed resistance to two or more antibiotics. Multi-drug resistance was found to increase significantly (*p* < 0.05) in the sites downstream of a WWTP for isolates resistant to ≥2, ≥3, and ≥4 agents. Other studies have observed high rates in the development of multidrug resistance in *E. coli* isolates in WWTP processes (Korzeniewska et al., 2013; Amador et al., 2015), found to be primarily driven by the transfer of conjugative plasmids (da Silva et al., 2006). A number of WWTP disinfection practices had negligible effects on reducing rates of multidrug resistant bacteria, and in a number of cases increasing it (Bouki et al., 2013). Even if the WWTP effluents in this study had considerably low levels of multidrug resistant bacteria, in other studies (Rizzo et al., 2013) these low concentrations have been shown to persist and propagate in the environment once discharged. ARG not necessarily bound to culturable organisms are also likely escaping treatment processes and contributing to the development of multidrug resistance (Kümmerer, 2009). Resistance to cefoperazone, sulfamethoxazole, ciprofloxacin, and gentamycin was more frequently found in multidrug resistant isolates, and rarely as the only type of resistance. This suggests that resistance to these agents is either driven by similar modes of defense coded by resistance genes to other agents, or that the acquisition of resistance to these agents usually occurs in tandem with other antibiotic resistances.

The high rates of resistance to cephalothin across all six sampling sites inflated multi-drug resistance rates, present in 95% of the 248 isolates resistant to at least one antibiotic. This increases the importance of the multidrug resistance classifications of isolates resistant to three or more and four or more agents, due to cephalothin resistance effectively acting as a resistance baseline for this data set. While the strictest definition for multidrug resistance is resistant to two or more agents, “resistance to three or more classes” has become increasingly standard for defining multidrug resistance in Gram-positive and Gram-negative bacteria (Magiorakos et al., 2012). Still, a substantial number, 9% of all 280 isolates, expressed resistance to at least 3 antibiotics. This rate is more in line with other reports of the prevalence of multidrug resistant *E. coli* in surface waters (Blaak et al., 2015), though these rates likely differ considerably as a function of antibiotics tested and sampling site. A large majority (86%) of these isolates were collected downstream of a WWTP, and all significant increases in rates of isolate resistance to three or more agents occurred when comparing an upstream site to a downstream site. While some degree of multidrug resistance appears to exist naturally, the results suggest that WWTPs in the watershed might be contributing significantly to multidrug resistant bacteria in the surface water.

### HPC-Ab

A significant increase in the concentrations of HPC-Ab downstream of WWTP discharge was found for all four agents tested against the total HPC community. Heterotrophic bacteria populations are diverse and possess a considerable amount of intrinsic variability in the way they occur and interact in the environment (Garcia-Armisen et al., 2013). By normalizing the abundance of HPC-Ab in the study area to the total heterotrophic bacteria population, a better understanding can be made concerning the extent of HPC-Ab relative to total numbers. Unfortunately, this diversity also makes it difficult to establish a reliable standard for which to compare resistance rates against. Additionally, the levels of HPC-Am, -Cpr, and -Te bacteria were generally confined to a range of 1–10% of the total heterotrophic community when compared to the control, though in some instances spiking to between 20 and 40% of the total population. However, these large spikes in the ratios of HPC-Ab to the control CFU were generally due to significantly lower counts in the control during a sampling event or at a sampling site and not because the CFU of HPC-Ab increased. In contrast, HPC-Su were frequently found to represent from 20 to 80% of the total heterotrophic population, ratios significantly (*p* < 0.001) higher than all other HPC-Ab. This same trend in HPC-Su was found to exist throughout numerous processes sampled in a municipal wastewater treatment plant (Gao et al., 2012), also finding that while the total abundance of resistant bacteria were reduced in the effluent, that reduction was consistent with the reduction in total HPC-Ab populations. The similarities in HPC-Su observed in the downstream sites in this study may indicate contribution of resistance traits originating from WWTP effluent.

The occurrence of a significant increase in the concentrations of HPC-Te bacteria downstream of WWTPs in the total heterotrophic populations appears to contradict the findings of Sullivan and Karthikeyan (2012), research also conducted in the Carters Creek watershed. Sullivan and Karthikeyan (2012) found no effect of WWTP location on the prevalence of HPC-Te in surface water, but did see an increase in the abundance of tetracycline ARG. While molar concentrations of tetracycline used in both studies were similar, the discrepancy might be explained by differences in the cultivation media: Sullivan and Karthikeyan (2012) used nutrient-rich agar and this study used nutrient-limited R2A agar. Differences in cultivation media can significantly affect the counts of culturable HPC even from identical samples (Garcia-Armisen et al., 2013). Sullivan and Karthikeyan (2012) also found no seasonal variability in the occurrence of tetracycline resistant genes or bacteria.

While there was a significant increase in the abundance of HPC-Ab in the downstream sites, there was no significant increase when the concentrations were normalized to total heterotrophic bacteria with no antibiotic in the cultivation media. This indicates that while the total amount of resistant bacteria is increasing downstream through the watershed, it is increasing proportionately with the total population. This can be due to several plausible reasons. Viable bacteria from treated effluents may be a reason to increase total abundance without increasing normalized rates of resistance in the watershed. Suspended solids, dissolved organic carbon, and nutrients in WW effluents may facilitate the growth of pre-existing HPC-Ab downstream. Favorable growth conditions and increased total heterotrophic population proportionately increased the abundance of resistant bacteria.

Total heterotrophic population CFUs on control plates during antimicrobial studies can vary dramatically (3 orders of magnitude) (Pei et al., 2006), making it difficult to normalize results of antibiotic bacteria within the population. Additionally, the methodology used to capture HPC-Ab is known to capture both intrinsic and acquired resistance traits (Brooks et al., 2007); thus, these values may represent an over representation of the antibiotic resistant population.

### Antibiotic Resistance Gene Prevalence

The scope of this project was not limited to only cultivable bacteria, the study also evaluated the prevalence of ARG by qPCR methods. Both the cultivation-based approach and the qPCR approach reveal a major difference in the AMR bacterial numbers in the downstream vs. the upstream sites. The detected dominance of tetracycline resistant genes is not surprising as several other studies that focused on the distribution of ARG in urban waters, wastewaters, and WWTPs have previously found elevated levels of various tetracycline resistance genes (Szczepanowski et al., 2009; Gao et al., 2012; Brooks et al., 2014; Laht et al., 2014; Mao et al., 2015; Makowska et al., 2016; Ng et al., 2016). In addition, these results are in agreement with the culture-based results presented in the previous sections and an earlier study in the Carters Creek watershed where Sullivan et al. (2013) found an abundance of a variety tetracycline resistant genes and tetracycline resistant bacterial groups in several sampling sites within the watershed.

Class I integrons are genetic elements that are tangentially associated with the distribution of AMR and development of MDR among Gram-negative bacteria in a variety of environments (Cambray et al., 2010; Domingues et al., 2012; Gillings, 2014; Strugeon et al., 2016). The integron integrase gene (*intI1*) is the key fragment of the functional structure of Class I integrons as it is responsible for antibiotic resistance element-containing gene cassettes to be acquired, expressed and disseminated across bacterial species (Stokes and Hall, 1989; Collis and Hall, 1992; Domingues et al., 2012; Strugeon et al., 2016). Several previous studies have linked the presence of *intI1* with prevalence of MDR in different environments (Leverstein-van Hall et al., 2002; Stalder et al., 2012; Brooks et al., 2014; Hultman et al., 2018). Furthermore, an elevated quantity of Class I integrase genes has been found to be present in studies that examined the occurrence of *intI1* specifically in WWTPs (Makowska et al., 2016; McConell, 2017) and further associated with anthropogenic environmental influence (Gillings et al., 2015). Therefore, the considerably high abundance of the *intI1* genes observed in all sites within this study is consistent with the observation of increased instances of AMR and MDR bacterial population in this site. It is important to note that we did not measure 16S rRNA while we measured ARG. Thus, we were not able to normalize our data to 16S rRNA which would provide for proportional context to the data set.

## Conclusion

Results from this study find a considerably greater AMR and MDR in the downstream sites of the WWTPs carrying the wastewater effluents compared to the upstream sites. Downstream sites showed an increased resistance to the antibiotics ampicillin, ciprofloxacin, cefoperazone, sulfamethoxazole, and tetracycline, and were more often resistant to a higher number of different antibiotics. These effects were mirrored in the total HPC-Ab community, with a significant increase in the abundance of HPC-Am, -Cpr, -Su, and -Te bacteria in the surface water downstream of WWTP discharge points Quantitative PCR analysis of eight ARG in the samples also revealed similar results. In addition, we also noted in this study that the class 1 integron integrase gene, previously widely reasoned in similar studies to be associated with mobile genetic elements responsible for the movement of AMR and MDR between bacterial species in several environments, was detected at significantly high concentration in all sites at all times. We also detected a markedly higher abundance of most of the tested genes, including the Class I integrons, in the site immediately downstream of the WWTP.

Antibiotic resistance and increased rates of resistance can be attributed by several factors, including WWTP discharges as reported here. A more specific future investigation and more constrained system focusing on the inflows, outflows, and process components at the WWTPs would be beneficial in determining the extent of its contribution to resistance in the environment. Occurrence and persistence of antibiotic resistance and maintenance of the resistome are complex to describe in natural settings such as watersheds with varying hydrology, land use changes, and anthropogenic activities. Several watershed processes including overland runoff, stormwater outflow, and runoff from impermeable surfaces will affect the dissemination of antibiotic resistant bacteria. In watersheds, particularly in urban settings, WWTPs play a critical mitigation point for antibiotic resistance. Effective treatments should decrease further spread of resistance. It is important to note that only a particular fragment of potential ARG were tested in our analysis; additional insights could be obtained into these interpretations with a more extensive antibiotic “resistome” study. Further understanding of the interrelationships among ARB concentrations, ARG concentrations, antibiotic agents, microbial species, and environmental media will help modeling of antibiotic resistance transfer in terrestrial and aquatic environment.

## Data Availability Statement

The raw data supporting the conclusions of this article will be made available by the authors, without undue reservation.

## Author Contributions

MM, TG, EL, JB, and RK: conceptualization. MM, TG, EL, and JB: methodology. MM and EL: formal analysis and data curation. MM: writing—original draft preparation. MM, TG, JB, and RK: writing—review and editing. TG: supervision, project administration, and funding acquisition. All authors contributed to the article and approved the submitted version.

## Conflict of Interest

The authors declare that the research was conducted in the absence of any commercial or financial relationships that could be construed as a potential conflict of interest.
