# Supplementary material for: Increased Antimicrobial and Multidrug Resistance Downstream of Wastewater Treatment Plants in an Urban Watershed
Source: Front Microbiol. 2021 May 24;12:657353. doi: 10.3389/fmicb.2021.657353 (PMC8181147; doi:10.3389/fmicb.2021.657353)
Supplement: Supplementary file 1 [file Data_Sheet_1.PDF]

Supplementary Material

1. Supplementary Data

1.1. PCR Isolate Confirmation Results:

Isolates were confirmed as *E. coli* through PCR amplification of *uidA* with *E. coli*-specific primers and an expected amplicon size of approximately 400 bp (Bower *et al.*, 2005). Figure S1 shows the results of PCR amplicon gel electrophoresis of all 300 isolates. Out of the total 300 isolates collected for this study, 280 (93%) were confirmed as *E.coli*. Any isolate that returned negative results had a second cell suspension prepared at a higher concentration, and both the original suspension and concentrated suspension were run again to confirm the negative result. Positive and negative controls produced the expected results for each assay. Several isolates initially produced negative results, but when the additional cell suspension was made and a second reaction was performed to confirm the negative results, the isolates returned positive. The 20 isolates that were not confirmed were excluded from the results and statistical analysis of the study.

2. Supplementary Figures and Tables

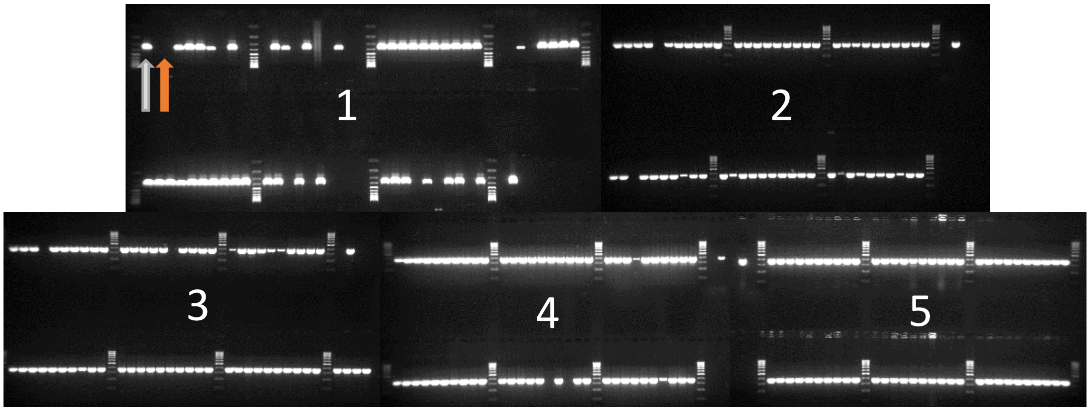

Figure S1: Results of PCR amplicon gel electrophoresis of *uidA* (~400 bp) for all 300 *E. coli* isolates obtained from sampling events 1 - 5. Green arrow = positive control; Red arrow = negative control.

Ampicillin		Sampling Site					
		1	2	3	4	5	6
Sampling Site	1		0.83	1.33	**2.81**	**4.24**	**8.33**
	2			3.64	**5.55**	**7.22**	**11.55**
	3				**0.29**	**0.89**	**3.25**
	4					0.18	1.67
	5						0.72
	6						

Sulfamethoxazole		Sampling Site					
		1	2	3	4	5	6
Sampling Site	1		0.91	0.95	**0.39**	**1.29**	**4.77**
	2			-	**1.91**	**3.11**	**6.66**
	3				**2.00**	**3.24**	**6.95**
	4					0.29	2.73
	5						1.27
	6						

Tetracycline		Sampling Site					
		1	2	3	4	5	6
Sampling Site	1		0.96	1.92	**4.30**	**7.17**	**10.45**
	2			4.38	**1.25**	**3.06**	**5.41**
	3				**8.57**	**11.76**	**15.08**
	4					0.47	1.67
	5						0.34
	6						

Gentamycin		Sampling Site					
		1	2	3	4	5	6
Sampling Site	1		2.78	2.91	**0.96**	**0.11**	**0.00**
	2			-	**0.95**	**2.05**	**2.73**
	3				**0.99**	**2.14**	**2.85**
	4					0.42	0.92
	5						0.10
	6						

Cefoperazone		Sampling Site					
		1	2	3	4	5	6
Sampling Site	1		0.91	0.95	**0.00**	**1.29**	**1.00**
	2			-	**0.95**	**3.11**	**2.73**
	3				**0.99**	**3.24**	**2.85**
	4					1.19	0.92
	5						0.03
	6						

Cephalothin		Sampling Site					
		1	2	3	4	5	6
Sampling Site	1		0.26	1.65	**3.01**	**0.26**	**0.33**
	2			0.57	**1.43**	**0.00**	**0.00**
	3				**0.20**	**0.57**	**0.53**
	4					1.43	1.42
	5						0.00
	6						

Ciprofloxacin		Sampling Site					
		1	2	3	4	5	6
Sampling Site	1		-	-	**2.13**	**3.45**	**7.38**
	2			-	**1.91**	**3.11**	**6.66**
	3				**2.00**	**3.24**	**6.95**
	4					0.29	2.73
	5						1.27
	6						

Imipenem		Sampling Site					
		1	2	3	4	5	6
Sampling Site	1		-	-	-	-	-
	2			-	-	-	-
	3				-	-	-
	4					-	-
	5						-
	6						

Figure S2: Chi-square test values for rates of isolate resistance between all sampling sites by antibiotic. Post-hoc multi-comparison tests between sites were significant at ($p < 0.003$) for test values > 7.5 (critical value for 1 degree of freedom). Values for which one site was upstream and the other was downstream are bolded. Shaded cells are tests that reported a significant difference in isolate resistance rates for that antibiotic. Cells with no value (-) indicate that no isolate resistance existed at one of the sites.

≥ 2 Agents	Sampling Site					
	1	2	3	4	5	6
Sampling Site 1		0.03	0.06	**2.23**	**6.66**	**8.11**
2			0.00	**2.59**	**6.98**	**8.37**
3				**2.88**	**7.51**	**8.98**
4					1.30	1.95
5						0.05
6						

≥ 3 Agents	Sampling Site					
	1	2	3	4	5	6
Sampling Site 1		0.91	0.95	**3.03**	**6.91**	**10.46**
2			-	**4.95**	**8.80**	**12.11**
3				**5.17**	**9.18**	**12.62**
4					1.06	2.99
5						0.47
6						

≥ 4 Agents	Sampling Site					
	1	2	3	4	5	6
Sampling Site 1		0.91	0.95	**1.13**	**3.34**	**5.83**
2			-	**2.90**	**5.30**	**7.69**
3				**3.03**	**5.53**	**8.03**
4					0.70	2.23
5						0.42
6						

Figure S3: Chi-square test values for rates of isolate resistance between all sampling sites by extent of multi-drug resistance. Post-hoc multi-comparison tests between sites were significant at ($p < 0.003$) for test values > 7.5 (critical value for 1 degree of freedom). Values for which one site was upstream and the other was downstream are bolded. Shaded cells are tests that reported a significant difference in multi-drug resistance rates for that site pairing. Cells with no value (-) indicate that no multi-drug resistance occurred at one of the sites.

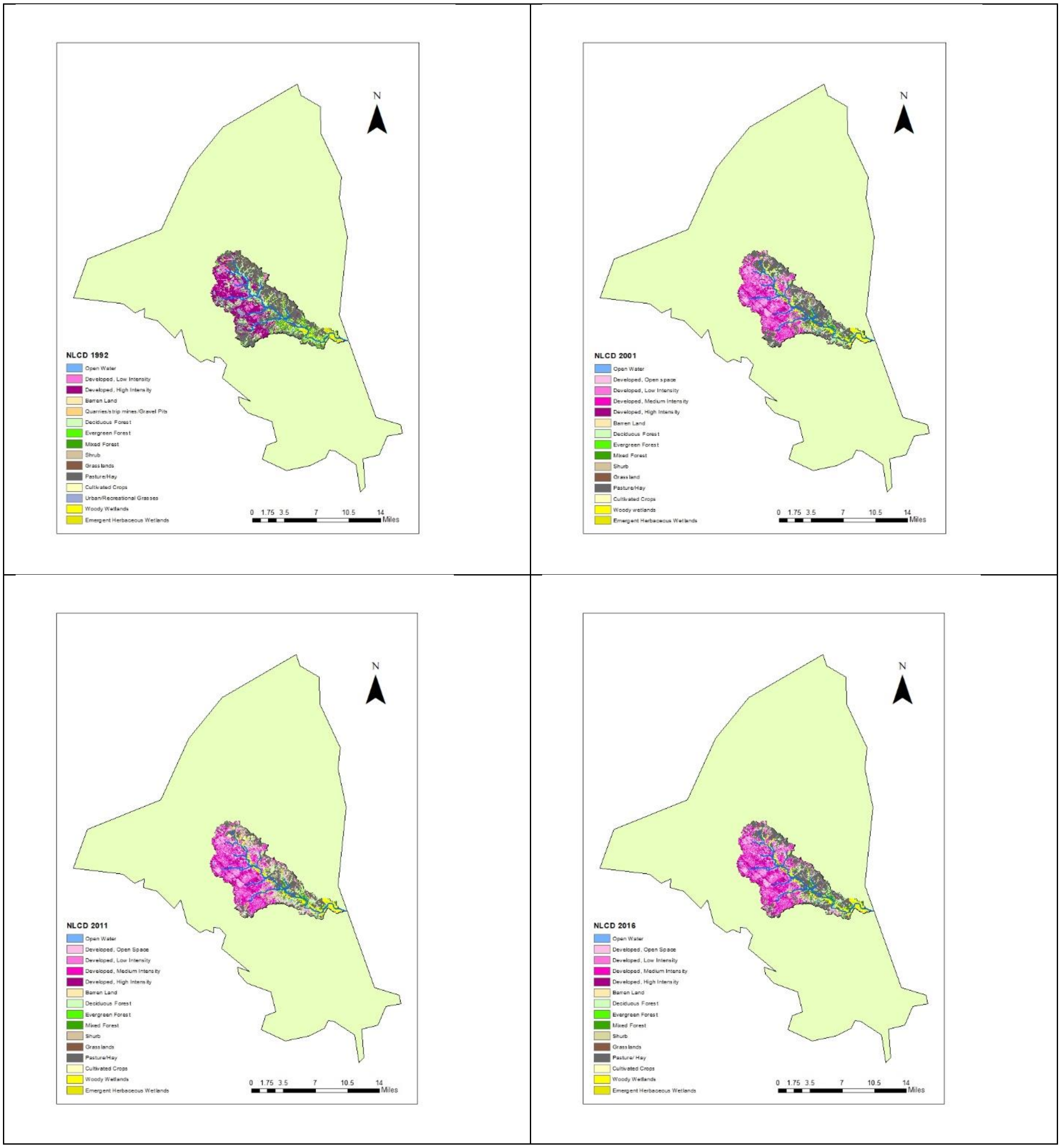

Figure S4: Land use changes in Carters Creek watershed over time (1992-2016) showing urban sprawl.

Table S1: Site Locations and Coordinates

Site Number	Description	Coordinates	Position in relation to WWTP
1	Carters Creek at Briarcrest Drive	30°40'04.3"N 96°19'13.2"W	Upstream
2	Burton Creek at Tanglewood Drive	30°38'26.8"N 96°20'06.6"W	
3	Carters Creek upstream of Burton Creek	30°38'40.3"N 96°18'43.2"W	
4	Burton Creek at Route 6, downstream of WWTP	30°38'39.2"N 96°18'50.4"W	Downstream
5	Carters Creek at Harvey Road	30°38'09.5"N 96°17'45.2"W	
6	Carters Creek at Bird Pond Road	30°36'10.6"N 96°15'00.1"W	

Table S2: Standards used in this study for quantitative PCR analyses

Standard for antibiotic resistant gene	Organism	Reference
Class I integron (*intI*)	Environmental *Escherichia coli* isolate	Brooks et al., 2014
Tetracycline (*tetA*)	Environmental *Escherichia coli* isolate	Brooks et al., 2014
Tetracycline (*tetW*)	Environmental *Bifidobacterium breve* isolate	Brooks lab
Ampicillin (*ampC*)	*Escherichia coli* ATCC 25922	ATCC® 25922™
Erythromycin (*ermA*)	Environmental *Staphylococcus spp.* isolate	Brooks et al., 2014
Methicillin (*mecA*)	*Staphylococcus aureus subsp. aureus*	ATCC® BAA1720™
Aminoglycoside (*aacA*)	Environmental *Lactobacillus brevis* isolate	Environmental isolate from Brooks lab
β-lactam (*blaTEM*)	*Pseudomonas aeruginosa* 27853	ATCC® 27853™

Table S3: Number of *E. coli* isolates (%) expressing resistance to antibiotics, by sampling site.

Antibiotic	Disc ID	Sampling Site						Total
		Upstream of any WWTP			Downstream of ≥ 1 WWTP			(n=280)
		1 (n=49)	2 (n=44)	3 (n=46)	4 (n=47)	5 (n=44)	6 (n=50)	
Tetracycline	TE-30	2 (4)	4 (9)	0 (0)	8 (17)	10 (23)	14 (28)	38 (14)
Ampicillin	AM-10	3 (6)	1 (2)	6 (13)	8 (17)	9 (20)	14 (28)	41 (15)
Cefoperazone	CFP-75	1 (2)	0 (0)	0 (0)	1 (2)	3 (7)	3 (5)	8 (3)
Ciprofloxacin	CiP-5	0 (0)	0 (0)	0 (0)	2 (4)	3 (7)	7 (14)	12 (4)
Sulfamethoxazole /Trimethoprim	SXT	1 (2)	0 (0)	0 (0)	2 (4)	3 (7)	7 (14)	13 (5)
Gentamycin	GM-10	2 (4)	0 (0)	0 (0)	1 (2)	2 (5)	3 (6)	8 (3)
Cephalothin	CF-30	44 (90)	38 (86)	37 (80)	36 (77)	38 (86)	43 (86)	236 (84)
Imipenem	IPM-10	0 (0)	0 (0)	0 (0)	0 (0)	0 (0)	0 (0)	0 (0)

Table S4: Log$_{10}$-transformed concentrations (log$_{10}$ CFU/mL) of HPC-Ab for each antibiotic by sampling event and sampling site.
HPC-None: No antibiotic added; HPC-Am: Ampicillin amended; HPC-Cpr: Ciprofloxacin amended; HPC-Te: Tetracycline amended; HPC-Su: Sulfamethoxazole amended

**Sampling Event**	**Date**	**AB**	**Concentration (log$_{10}$ CFU/mL) by Sampling Site**					
			1	2	3	4	5	6
**#1**	7/13/2015	HPC-None	3.05	6.08	3.52	5.28	4.78	4.21
		HPC-Am	2.67	3.22	3.03	4.20	3.85	2.70
		HPC-Cpr	< 1.52	2.12	1.52	2.52	2.52	2.12
		HPC-Te	1.82	< 1.52	2.52	3.82	3.10	2.12
		HPC-Su	2.88	3.88	2.82	4.29	3.70	3.15
**#2**	9/7/2015	HPC-None	5.10	5.29	4.59	5.41	5.23	5.43
		HPC-Am	3.52	3.70	3.29	4.04	3.90	3.65
		HPC-Cpr	3.82	3.70	3.75	4.11	4.18	4.26
		HPC-Te	2.52	2.90	2.22	3.47	2.90	2.87
		HPC-Su	3.88	4.18	3.53	4.70	4.32	4.52
**#3**	11/5/2015	HPC-None	4.32	4.46	4.29	5.17	4.59	5.17
		HPC-Am	2.82	3.17	2.85	3.56	3.85	3.56
		HPC-Cpr	3.56	3.48	3.59	3.94	4.08	3.94
		HPC-Te	2.52	2.52	2.37	2.87	3.22	2.87
		HPC-Su	4.12	3.87	3.64	4.66	4.19	4.66
**#4**	1/20/2016	HPC-None	5.17	5.50	5.05	5.21	5.17	5.20
		HPC-Am	2.70	4.37	2.94	3.99	4.00	3.64
		HPC-Cpr	3.32	3.78	3.69	3.46	3.72	3.50
		HPC-Te	1.52	2.85	1.82	3.18	3.11	2.67
		HPC-Su	3.34	2.85	3.43	4.56	4.73	4.48
**#5**	2/16/2016	HPC-None	5.04	5.16	4.58	5.30	5.27	5.41
		HPC-Am	2.37	4.04	2.12	4.22	4.17	3.77
		HPC-Cpr	< 1.52	1.82	< 1.52	3.79	2.88	2.97
		HPC-Te	2.43	2.92	2.56	3.64	3.65	3.48
		HPC-Su	3.99	4.52	3.22	5.20	4.85	4.43
**#6**	4/6/2016	HPC-None	4.70	5.09	4.17	5.65	5.39	5.39
		HPC-Am	3.22	3.43	3.11	4.41	4.41	4.03
		HPC-Cpr	1.52	2.22	< 1.52	3.90	3.87	3.21
		HPC-Te	3.56	3.15	3.17	3.95	3.92	3.95
		HPC-Su	2.90	3.52	2.43	4.95	4.64	4.01

Table S5: Mean antibiotic resistance gene copy numbers/ 100 mL surface water detected at Carters Creek upstream vs. downstream WWTP sites.

Antibiotic resistance genes	Upstream of WWTP (mean of all sampling events)	Downstream of WWTP (mean of all sampling events)	Site 4-downstream BCWWTP (mean of all sampling events)	p-values for each downstream/upstream pairwise comparison
Integrase *(intI1)*	1.25×10^{6}	2.51×10^{7}	5.50×10^{7}	p=< 0.0001
Tetracycline resistance *(tetW + tetA)*	7.33×10^{4}	9.89×10^{5}	5.31×10^{5}	*tetA* – p=< 0.0001<br>*tetW* – p=0.0007
Erythromycin resistance *(ermA)*	8.96×10^{3}	4.33×10^{5}	5.98×10^{5}	p=< 0.0001
Aminoglycoside resistance *(aacA)*	1.40×10^{4}	6.90×10^{4}	1.60×10^{5}	p=0.0603
TEM type β-lactamases (*blaTEM)*	9.65×10^{3}	3.13×10^{4}	7.33×10^{4}	p=0.0115
Ampicillin resistance *(ampC)*	4.68×10^{4}	4.87×10^{4}	6.77×10^{4}	p=0.7449
Methicillin resistance *(mecA)*	5.43×10^{2}	7.66×10^{2}	5.93×10^{2}	p=0.3681
